# Going with the flow: updating old techniques to gain insight into regional kidney hemodynamics

**DOI:** 10.14814/phy2.14103

**Published:** 2019-05-14

**Authors:** Paul M. O'Connor

**Affiliations:** ^1^ Department of Physiology Medical College of Georgia Augusta Georgia

Renal blood flow occurs both in series and parallel. While all the blood entering the kidney first enters the renal cortex, two distinct populations of nephrons give rise to remarkably different post‐glomerular capillary circulations. Superficial and mid‐cortical nephrons give rise to the cortical peritubular capillary network, a web of capillaries that supplies the cortical parenchyma and supports reabsorption of the bulk of the glomerular filtrate. In contrast, the majority of juxta‐medullary nephrons, whose afferent arterioles arise near the corticomedullary junction, give rise to vascular bundles containing descending vasa recta capillaries that supply blood to the renal medulla. Some of these descending vasa recta traverse all the way to the tip of the papilla, before looping around to giving rise to ascending vasa recta capillaries. Descending vasa recta on the periphery of these vascular bundles give rise to the dense plexus of capillaries that supply the medullary parenchyma. The relatively low rate of perfusion (less than 10% of total renal blood flow) and the looped structure of the medullary circulation limit the washout of solutes including NaCl and urea in this region, maximizing the ability to form concentrated urine.

Since the observation by Trueta et al. (1948) that renal medullary perfusion could be completely maintained, even when blood flow to the renal cortex is severely limited, the concept of functional intra‐renal distribution of blood flow has been of interest. Unlike most circulatory beds, renal perfusion is remarkably inert to metabolic stimuli such as hypoxemia (Leonard et al. 2001), a phenomenon which may be of benefit in the kidney's role as a crit meter. Many vasoactive hormones have distinct effects on regional kidney blood flow (Evans et al. 2004). Maintenance of renal medullary perfusion in response to vasoactive stimuli such as angiotensin II, that reduces renal cortical perfusion, may allow alterations in global renal hemodynamic forces that alter NaCl and water reabsorption without putting the less well perfused renal medullary region at risk of ischemia. Cowley et al. (1992) have championed the hypothesis that the rate of renal medullary perfusion acts as a signal for the pressure‐natriuresis mechanism, and therefore is critical to the maintenance of long‐term blood pressure. More recently, vascular rarefaction of the peritubular capillary network in the renal cortex has been suggested to underlie the development of renal hypoxia, which has been hypothesized to be a common mechanism in chronic kidney disease (Fine et al. 1998; Fine and Norman 2008). Vascular rarefaction in the renal outer‐medullary region following acute kidney injury has also been shown to be associated with the development of salt‐sensitive hypertension (Pechman et al. 2009). While much has been learned, there remains controversy regarding the functional role of regional kidney hemodynamic changes toward various disease states. Given the prevalence of diseases such as hypertension and chronic kidney disease, a greater understanding of the role of regional renal hemodynamics toward the development of these diseases is needed.

Much of the controversy regarding the role of renal hemodynamic changes toward disease likely arises from the difficulties associated with studying regional renal hemodynamic changes. While the kidney is perfused by a single artery, making measurement of total renal blood flow relatively simple, the parallel nature of the renal circulation makes quantifying regional hemodynamic changes difficult, particularly in the deeper less accessible areas such as the renal outer‐medulla. Further, most techniques utilized to quantify regional renal hemodynamics require prolonged anesthesia of the animal and exposure of the kidney, factors that undoubtedly have major effects of renal perfusion. Many techniques also lack the sensitivity to adequately quantify changes in regional kidney blood flow, particularly within the small post‐capillary vessels of the cortical peritubular circulation and post‐glomerular medullary capillaries.

The microfil technique, in which the renal circulation perfused with silicone rubber before dissolving the renal parenchyma, has provided an enormous amount of information regarding renal hemodynamics. Vascular casts of the renal circulation have been utilized to visualize the complex tubular vascular relationships that support renal function as well as understand the actions of vasoactive stimuli on glomerular afferent and efferent arteriole diameters (Denton et al. 1992). Vascular casts have also been utilized to demonstrate changes in regional kidney perfusion in response to vasoactive stimuli or following pathological interventions such as acute kidney injury (Mason et al. 1984). A major limitation of the microfil technique has been incomplete perfusion of the post‐glomerular capillary vasculature. Given the significant interest in quantifying the post‐glomerular capillary circulation, this limitation represents a major obstacle to understanding the functional role of regional kidney hemodynamics in disease states.

In this issue, Fan et al. (2019) describe a modified technique to fill the renal vasculature with a silicon rubber. Importantly, by decreasing the viscosity of the microfil solution and degassing this solution prior to perfusing the kidney, Fan et al. demonstrate that they are able to reproducibly fill and image vessels down to ~10 micrometers in diameter in the post‐glomerular circulation of rats. This includes the peritubular and vasa recta capillary beds of which there is currently great interest. Importantly, Fan et al. (2019) demonstrate that this improved in vivo filling technique does not appear alter arterial diameters or the pattern of blood flow (as assessed using video microscopy). Further, changes in the distribution of regional kidney blood flow in response to vasoactive maneuvres assessed using this technique are similar to those reported by previous studies utilizing a variety of alternative approaches. While all techniques are subject to limitations, Fan et al.'s demonstration of an improved microvascular filling technique that enables more complete filing of the small renal microvasculature is likely to enhance our understanding of regional kidney hemodynamics and the role that changes in the renal post‐glomerular circulation plays in the pathology of a variety of disease states.
